# Assessing medical student cultural competence: what really matters

**DOI:** 10.5116/ijme.578b.687c

**Published:** 2016-07-30

**Authors:** Windsor W. Sherrill, Rachel M. Mayo, Khoa D. Truong, Anne P. Pribonic, Christine A. Schalkoff

**Affiliations:** 1 Department of Public Health Sciences, Clemson University, Clemson, SC, USA; 2 School of Health Research, Clemson University, Clemson, SC, USA

**Keywords:** Cultural competence, medical education, Latino, medical students

## Abstract

**Objectives:**

The study aimed to explore medical students’ attitudes and beliefs toward Latino patients, specifically: to assess students’ levels of knowledge, cultural competence, and comfort with Latinos; to determine students’ exposure to and previous experience with Latinos; and to evaluate whether factors such as study abroad, living abroad, previous clinical experience with Latinos, and language proficiency predict Latino knowledge, cultural competence, and comfort with Latinos.

**Methods:**

This study utilized a cross-sectional survey design. Participants were third and fourth year medical students at three medical schools in the Southeastern United States. Three composite measures: Latino knowledge, Cultural competence, and Comfort with Latino patients, were predicted in a multivariate regression model including individual sociodemographic characteristics and past clinical or social experience with Latinos.

**Results:**

A total of 170 medical students completed the survey (43% response rate). Spanish language proficiency was a statistically significant predictor (t_(131)_=2.72, p<0.05) of Latino knowledge. Social interaction with Latinos in the past year (t_(126)_=3.09, p<0.01), ever having lived in a Spanish-speaking country (t_(126)_=2.86,  p<0.01), and Spanish language proficiency (t_(126)_=3.28, p<0.01) independently predicted cultural competence. Previous clinical experience with Latinos was not significantly associated with the three composite dependent variables, and comfort with Latino patients was not significantly predicted by any of the six Latino-related explanatory variables.

**Conclusions:**

Factors prior to medical school matriculation and during medical education may contribute to increased cultural competence and comfort with multicultural patients. Cultural patient-partner programs may be an effective way to increase cultural competence within the confines of medical school curricula.

## Introduction

According to a 2014 US Census Bureau report, the Latino population constitutes approximately 17% of the total  population, and is predicted to grow to 31% by 2060.^1^  This growing Latino population presents a unique set of healthcare-related challenges, both for assimilating immigrants and the adapting healthcare system.^2^  Obstacles to healthcare are especially evident in healthcare delivery to Latinos, where patients face challenges in accessing quality care services.^3^  Patient-physician interaction and perceived language and cultural barriers are often the most cited causes for reduced care quality and satisfaction.^3^^,^^4^  Cultural barriers often exist when there is a perceived lack of cultural competence- the provider’s ability to recognize and respond to health-related beliefs and cultural values, especially in regard to disease and treatment.^5^

A lack of cultural competence in Latino patient-provider relations leaves many Latino patients feeling dissatisfied with health services, less confident in their providers, and less likely to adhere to treatment plans.^6^^-^^8^  In a Commonwealth Fund study, 15% fewer Latinos reported that they had a great deal of confidence in their doctors than white patients posed the same question.  In addition, 26% of Latinos reported that physician advice conflicted with their beliefs. This distrust and lack of acknowledgement of differences could contribute to the finding that 22% of Latinos reported not following provider advice.^6^

Culturally competent graduates from health-related education programs are a necessity for a health workforce prepared to address illness in the context in which it is experienced by patients and families. The Association of American Medical Colleges’ (AAMC) recent memorandum “Cultural Competence Education for Students in Medicine and Public Health” organizes competencies and learning objectives by knowledge, skill, and attitudes, citing the need to practice in a culturally competent manner and its benefit in improving health outcomes and reducing health disparities.^9^

Research suggests that medical students and physicians are aware of the need for culturally competent care, even if they do not feel prepared to provide it. In a study of resident physicians, residents acknowledged the importance of obtaining skills and training in cross-cultural care, and noted that less-than-optimal skills and training could negatively impact the physician-patient relationship.^10^  In a separate study, 96% of providers agreed that it is moderately or very important to address cultural issues when providing care. However, 25% of providers believed they were not prepared to address health needs of new immigrants.^11^ Disparity exists between concern for culturally competent care and comfort treating diverse populations.

This widespread deficiency in culturally competent care among healthcare providers may stem from two issues facing the healthcare system:  a disconnect between providers and the communities they serve, as well as shortfalls in the medical education system in providing cultural competence education strategies. The former may be partially attributed to the large disparity between the proportion of Latinos in the general population compared to the proportion of Latino physicians.^12^  Between 2005 and 2012, the percentage of Latinos in the  US grew from 14.4% to 17%.^13^  In contrast, in the same time period, the percentage of matriculating Latino medical students remained relatively constant (7.7% in 2005, 7.9% in 2012) and was substantially lower than the percent of Latinos in the general  population.^14^  For South Carolina and Georgia (states which served as sources of data for this study), Latinos represent 5% and 9% of the state population, while Latino medical students represent 2.7% and 2.8%, respectively.^14^  Disproportionality in the number of Latinos in the provider population compared to the overall population of patients limits the cultural diversity of the healthcare workforce, making it harder for patients to feel connected with providers.

These challenges may be successfully addressed through the medical education system’s cultural education strategies. Accreditation organizations have acknowledged the importance of cultural competency in medical education and have made efforts to promote it in medical training. The Liaison Committee on Medical Education (LCME) and the Accreditation Council for Graduate Medical Education (ACGME) have implemented policies demonstrating a commitment to ending existing disparities through progressive accreditation standards.^15^^,^^16^  The LCME requires that medical schools help students become self- aware concerning their own cultural biases. Medical education must include training in the assessment of how people of other belief systems perceive healthcare and the American healthcare system. The ACGME requires that residency programs teach providers in training to communicate well enough to collaborate with patients and successfully share information while overcoming hurdles presented by cultural diversity (including religion, ethnicity, gender, or sexual orientation) among patients.

The education of healthcare providers may prove to be an opportunity for well-placed intervention. AAMC surveys of matriculating medical students from 2009 to 2011 regarding anticipated training found a significant receptiveness to cultural competency advancement.^14^  Yet with such a multifaceted issue as culturally competent care for diverse populations, it is difficult to determine the most appropriate intervention strategies to target in the medical education system. Several studies have examined the effects of cultural competence interventions for medical school students, with largely positive results. These interventions have taken the form of international immersion experiences, service-learning projects, or classroom-based modules and lectures. Campbell et al. evaluated the effect of an international immersion/medical mission experience on cultural competence. Students self-reported personal growth, an increased appreciation for the impact of culture on health, an increased ability to communicate with patients of different cultures, and a deeper understanding of disparities in healthcare.^17^  Musolino and colleagues’ examination of a “Cultural Competency and Mutual Respect” program at the University of Utah indicated that student scores on the Inventory for Assessing the Process of Cultural Competence Among Healthcare Professionals-Revised (IAPCC-R) survey increased after the completion of classroom modules centered on cultural competence,^18^ and a 2006 evaluation of a three-hour cultural proficiency workshop also demonstrated movement towards increased cultural competency.^19^  Carpenter et al.’s randomized trial compared a web-based cultural competence curriculum to a traditional lecture format, and found the web-based module to be just as effective as the lecture in increasing cultural competence.^20^  However, these studies are still limited by a lack of consistent and rigorous evaluation methods, as well as problems inherent in the self-reporting of students.^21^

In particular, discrepancies between students’ self-reported levels of confidence in cultural competence and their actual levels of cultural knowledge and cultural competency skills are notable. Two studies have found that students have relatively low knowledge of the skills required for caring for Latino patients, and results of additional studies confirm low baseline cultural competence knowledge of medical students.^22^^-^^24^  In spite of this, students reported high levels of comfort with diverse populations.^25^  A 2014 study of medical students reported a weak association between actual cultural competence and knowledge and self-perceived ability in those areas – students had low scores on knowledge, yet perceived themselves to be more culturally competent.^26^  Additionally, researchers have suggested that in cases where students complete a survey to measure cultural competence, they may demonstrate social response bias, answering questions in a way that reflects what they think researchers or superiors want to hear, not how they actually feel.^21^^,^^27^

More research is needed to evaluate the effectiveness of cultural competency courses as well as specific predictors of cultural competency among medical students. This study focuses on predictors of cultural competency and comfort with Latino patients, as well as objective measures of knowledge of the Latino population among providers in training.  The study attempts to answer the following questions:

1.     What are medical students’ levels of knowledge, cultural competence, and comfort with Latinos?

2.     What are medical students’ exposure to and previous experience with Latinos?

3.     Do factors such as study abroad, living abroad, previous clinical experience with Latinos, and language proficiency predict Latino knowledge, cultural competence, and comfort with Latinos?

## Methods

### Study design and participants

This study utilized a cross-sectional survey design.  Participants were all third and fourth year nursing and medical students at four nursing and three medical schools in the Southeastern United States.

### Instrument development

The initial step in survey development was a comprehensive review of the psychosocial, educational, and behavior-related literature related to healthcare for Latinos. Efforts were made to identify relevant existing scales and questionnaires applicable to the study. A survey instrument was designed based on van Ryn and Fu’s theoretical model, which suggested that patients’ race/ethnicity influence providers’ beliefs, and providers’ beliefs affect their clinical decision-making and ultimately contribute to racial inequalities in healthcare for Latinos.^28^ A new survey, the Medical and Nursing Student Readiness to Treat Latino Patients (MaNSRT) was developed. After approval was obtained from the Clemson University Institutional Review Board, a pilot survey with the proposed items was administered to a convenience sample of 65 students.

Copies of the survey were sent to two Latino health expert reviewers for assistance in the relevance, usefulness, and wording of each item. Comments from the Latino health experts helped eliminate excessively vague and superfluous items, directed the revision of item language for clarity and content, and suggested pertinent follow-up questions to items. Expert review and initial pilot work resulted in significant revisions to the proposed instrument. Focus groups of potential respondents were key to informing survey development. Working with educational programs, students from two medical schools and four nursing schools were recruited to participate in 90-minute focus groups to collect information regarding experience with Latino patients and exposure to cultural competence and cancer-related education as well as to evaluate planned survey items. Emerging themes were identified and compared to the study’s conceptual model for construct validity. Following the focus groups, the research team consulted with psychometric experts to incorporate focus group feedback as well as assess formatting of the survey, including “soft” prompts to facilitate capture of data, balanced stem wording to prevent respondent bias, and the use of item-specific survey items rather than Likert Scale items. The online survey methods team members then began the process of survey construction and formatting of the instrument for the on-line environment.

To obtain preliminary information regarding the item response distributions and construct validity, the revised online survey was pilot-tested among a group of medical and nursing students. Analysis of this data provided final decisions regarding items for elimination from the survey and composite variables as well as a safeguard against major blunders associated with the survey format, content, and readability.

The final MaNSRT survey included 112 items, which were used to construct composite measures. Composite measures were developed by combining groups of survey items related to specific constructs. Constructs included: Latino knowledge; Attitude and beliefs about Latinos; Cultural competence; Comfort with Latino patients; and Previous Latino experience. For example, in the composite measure of Latino knowledge, survey participants were asked “True/False: Hispanics/Latinos are less likely to engage in high-risk sexual behavior than non-Hispanics.” An item included within the composite measure of Comfort with Latino patients was the question “Do you find it difficult to communicate well enough with Hispanic/Latino patients to assess medical conditions adequately?” For the composite measure of Cultural competence, questions such as “On a scale of 1 to 5, how important do you feel it is for providers to consider the patient’s culture when providing care?” were asked. Additional questions were reported by Mayo et al. in 2014.^28^

The final survey included 6 questions related to Latino knowledge, 19 questions related to Cultural competence, and 15 questions related to Comfort with Latino patients. Responses to the questions were formatted in such a way that higher scores (1: lowest, 5: highest) indicate better knowledge, more positive attitudes, or higher comfort with Latinos. All measurement items were self-rating. The respondents were also asked to provide sociodemographic characteristics including year in program, Spanish language proficiency, and related previous Latino experiences (e.g., having visited a Spanish-speaking country). A combined mean score for each composite measure was calculated, and the Cronbach’s alpha coefficient was computed to measure the internal consistency of the composite measures: Latino knowledge (6-30 range), Cultural competence (19-95 range), and Comfort with Latino patients (15-75 range). It was found that 3 composite measures were important to the study.

### Data collection methods

The survey was administered on-line through a secure link sent to all eligible students. The data presented in this paper include medical students only; nursing student results were reported separately in 2014.^29^ Third and fourth year medical students were recruited through the Associate Dean’s office at each university. The response rate was 43%, and 170 medical student responses were included in analysis. Table 1 provides the descriptive statistics of the medical students in the sample.

**Table 1:** Descriptive statistics of sample students (N=170)

Sociodemographic characteristics	n	%
Gender		
Female	94	55.29
Male	76	44.71
Race		
Non-Hispanic White	116	68.24
Black	12	7.06
Hispanic	8	4.71
Other	34	20.00
Age in years (Mean & SD)	25.81	2.24
Latino exposure and experience		
Social interaction with Latinos in the past year	147	88.00
Have visited a Spanish-speaking country	95	55.88
Ever lived in a Spanish-speaking country	13	7.65
Had some clinical experience with Latinos	157	94.58
Completed a population health class	62	36.47
Some Spanish proficiency	87	51.79

Approximately 55% of the students were female and 45% male. Less than 5% of respondents were Latino and approximately 70% were White. The average age of the students was 25.8 years. The study sample contained a slightly higher Latino population compared to average medical school demographics. Approximately 4.7% of participating students identified themselves as Latino, compared to approximately 2.7% of the Latino medical student body found in South Carolina and Georgia medical schools. This slight increase could be attributed to a self-selection due to personal interest in the survey topic. The majority of students had some interaction with Latinos in the past 12 months, more than half had visited a Spanish-speaking country, 7.7% had lived in a Spanish-speaking country, and almost all had some clinical experience with Latinos. Half of the students had some Spanish language proficiency, and about a third had completed a Population Health course.

### Statistical analysis

A multivariable regression model was used to identify relationships between the variables of interest. Each of the three composite measures (Latino knowledge, Cultural competence, and Comfort with Latino patients) was treated as a dependent variable in a regression model in which the predictor variables included gender (male as the reference group), race (White, African-American, Other, and Hispanic as the reference group), age (in years), and six binary variables: Social interaction with Latinos in the past year; Visited a Spanish-speaking country; Ever lived in a Spanish-speaking country; Clinical experience with Latinos; Completed a population health class; and some Spanish proficiency. A set of dummy variables to account for the school “fixed effect” was also included in the regression model.

## Results

The mean, standard deviation, minimum and maximum scores for the three composite multi-item measures of Latino knowledge, Cultural competence, and Comfort with Latino patients were calculated. Latino knowledge had 6 items with a five-point Likert Scale (for a range of 6 to 30 total points possible), and the Cronbach's alpha was 0.8764. The mean score of Latino knowledge was 15.35 with a standard deviation of 3.65. Cultural competence had 19 five-point items (for a range of 19 to 95 total points possible), and its Cronbach's alpha was 0.8430. The mean score was 59.99 and standard deviation was 8.10. Lastly, Comfort with Latino patients had 15 five-point items (for a range of 15 to 75 total points possible) with the Cronbach's alpha of 0.8964, mean score of 45.19, and standard deviation of 7.61.

As shown, having some Spanish proficiency was a statistically significant predictor (t=2.72, p<0.05) of Latino knowledge. Social interaction with Latinos in the past year (t=3.09, p<0.01), ever having lived in a Spanish-speaking country (t=2.86, p<0.01), and Spanish language proficiency (t=3.28,  p<0.01) independently predicted cultural competence. Interestingly, previous Latino clinical experience was not significantly associated with any of the three dependent variables and Comfort with Latino patients was not significantly predicted by any of the 6 Latino-related explanatory variables. One single consistent (and obvious) finding related to sociodemographic characteristics, was that, compared to White, Black, and other Race/Ethnicity, Latino was significantly associated with a higher score for each of the three dependent variables (Latino Knowledge, Cultural Competency, Comfort with Latinos).Table 2 presents results from the multivariable regression models. The set of explanatory variables presented in the first column was used for each regression model with one of the three dependent variables on the top row.

As shown, having some Spanish proficiency was a statistically significant predictor (t_(__131)_=2.72, p<0.05) of Latino knowledge. Social interaction with Latinos in the past year (t_(__126)_=3.09, p<0.01), ever having lived in a Spanish-speaking country (t_(126)_=2.86, p<0.01), and Spanish language proficiency (t_(126)_=3.28,  p<0.01) independently predicted cultural competence. Interestingly, previous Latino clinical experience was not significantly associated with any of the three dependent variables and Comfort with Latino patients was not significantly predicted by any of the 6 Latino-related explanatory variables. One single consistent (and obvious) finding related to sociodemographic characteristics, was that, compared to White, Black, and other Race/Ethnicity, Latino was significantly associated with a higher score for each of the three dependent variables (Latino Knowledge, Cultural Competency, Comfort with Latinos).

**Table 2:** Predictors of Latino knowledge, cultural competence, and comfort with Latino patients

Predictor Variables	Latino knowledge (n=145)			Cultural competence (n=140)			Comfort w/ Latino patients (n=142)		
	Coef.	*t*		Coef.	*t*		Coef.	*t*	
Social interaction with Latinos in the past year	1.03	1.06		6.00	3.09	**	-0.41	-0.19	
Have visited a Spanish-speaking country	0.85	1.11		0.99	0.64		0.45	0.28	
Ever lived in a Spanish-speaking country	1.12	1.02		6.22	2.86	**	3.61	1.57	
Had some clinical experience with Latinos	1.35	1.04		2.21	0.74		5.66	1.87	
Completed a population health class	-0.24	-0.31		-1.04	-0.66		1.28	0.79	
Some Spanish proficiency	1.87	2.72	**	4.57	3.28	**	2.43	1.69	
Female^1^	-0.53	-0.86		1.09	0.89		-0.56	-0.43	
White^2^	-3.70	-2.03	*	-9.19	-2.57	*	-11.26	-2.98	**
Black^2^	-4.00	-1.87		-14.25	-3.39	**	-16.11	-3.63	**
Other^2^	-4.63	-2.46	*	-10.75	-2.90	**	-12.24	-3.12	**
Age	0.09	0.71		0.40	1.53		0.13	0.48	
School 2^3^	0.06	0.08		-2.76	-1.86		-1.20	-0.75	
School 3^3^	1.14	0.94		-2.14	-0.86		0.66	0.26	
Constant	13.29	3.28		50.64	6.34		47.34	5.55	

^1^Male is the reference group; ^2^Hispanic is the reference group; ^3^School 1 is the reference group

*p < 0.5; **p < 0.01

## Discussion

Multivariate regression findings suggest that factors prior to medical school matriculation and during medical education may contribute to higher levels of Latino knowledge, cultural competency, and comfort with Latino patients. Of factors that were possible predictors of Latino knowledge, only having Spanish proficiency was statistically significant. Spanish proficiency was also predictive of cultural competence, although not of comfort with Latino patients. Similarly, having lived in a Spanish-speaking country was associated with cultural competence but not comfort with Latino patients. Cultural competence was also predicted by social interaction with Latinos in the past year.

Perhaps most surprisingly, clinical experience with Latinos was not predictive of Latino knowledge, cultural competence, or comfort with Latino patients. Mayo et al. noted that medical students reported their exposure to Latino patients generally in the context of primary and emergency care settings.^30^ The limited scope of cultural  experience among medical students could account for the low predictive value of previous clinical experience on Latino knowledge, cultural competency, and comfort with Latino patients.

As the proportion of Latinos in the US continues to increase, the need for a population of medical providers that can provide culturally competent care also increases. As discussed, the proportion of Latino medical students has not increased in recent years, indicating that the population of medical providers is not reflective of the general population. While medical schools may already be recruiting Latino students in an effort to decrease this disparity, efforts should also be made to help the remaining non-Latino medical students become as adept as possible at delivering culturally competent care.

Findings from our empirical work identify a set of attributes which can be influenced via educational programs, but not without challenges. Spanish language proficiency was associated with both Latino knowledge and cultural competence. Factors such as clinical experience and coursework were associated with the outcomes of interest (cultural competence, comfort with Latinos) in the expected direction, but the relationships were not statistically significant. Explanations may include limited sample size, small effect size, and limited ability to capture the key constructs through the online survey. Insignificance of associations may also suggest that a “quick fix” to impact cultural competence may not work. Obtaining clinical skills and experiences with Latinos requires considerable training and available clinical training with a population group. Some training related to Latinos is beneficial (and was associated with positive outcomes on the variables of interest), but limited exposure to specific patient groups may not be enough for sustained impact on future practice or clinical competence. The study findings suggest that “dosage,” or the length of exposure and training provided, may be an important factor impacting provider practice and perception.

However, the implementation of the programs required to increase the attributes that were associated with increased cultural competence (Spanish language skills, social interaction with Latinos, living in a Spanish-speaking country) may not be practical for the medical school curriculum. For example, it is unlikely that medical schools could offer a required study-abroad program for students, given the already-extensive curriculum requirements and course schedule.

An alternative solution for medical educational programs, given the empirical evidence, is to intentionally seek Latino interests and experience among prospective students. Educational programs can provide for such experiences after matriculation, but giving weight to candidates with previous Latino interest and background can be a cost-effective solution. Programs may consider recruiting students with previous experiences such as study abroad, language proficiency, or living in a Spanish-speaking country rather than only attempting to incorporate these initiatives within training programs. However, this controversial approach is also not an ideal one – it runs the risk of creating medical school admissions criteria that are unfairly biased against students without the resources to pursue study abroad or similar experiences prior to medical school.

Given the challenges and problems associated with both of the previously mentioned approaches, we suggest a third, novel approach. While it may not be practical to implement study-abroad or Spanish language programs, nor ethical to screen for students with prior experience in a Spanish-speaking country, we believe that there is a third way to increase the cultural competency of medical students within the established framework of the medical school curriculum. Some medical schools, in accordance with a shift towards patient-centered care, have implemented patient-partner programs, in which medical students and patients with chronic diseases are paired together in order to increase medical student knowledge and positive attitudes towards patients.^31^^,^^32^  We suggest that this model could be adapted for increasing medical student cultural competence through the introduction of a “cultural partner.” In this model, medical students could be matched with a Latino patient-partner throughout their medical school career. Through this partnership, students would potentially be able to increase some of the attributes which were predictors of cultural competence in our study, such as Spanish language skills and social interaction with Latinos. Additionally, this model could be adapted according to geographic and population demographic needs – cultural partners could be patients of various ethnic backgrounds.

### Study limitations

There are several challenges associated with the data and findings from the survey.  Sample size is a clear limitation. Significant associations may emerge with a larger sample. Students were self-reporting, and many of the items are likely to be subject to social desirability response bias. Student providers are increasingly aware of the importance of cultural competence, so they are likely to answer questions in ways they perceive as being more acceptable to their programs, regardless of actual attitudes, beliefs and behaviors.

Finally, the respondents to the study were third and fourth year medical students with a mean age of 25.8 years. Some of the constructs assessed in the survey may be skills that would develop as they become more seasoned providers and have more clinical experience. In many cases, they are self-reporting on factors of practice that are not yet relevant or developed, such as specific clinical skills and competencies. As such, they may be either overly – or under-confident of their skills and ability.

## Conclusions

The Institute of Medicine (IOM) continues to provide impetus for change in medical education as a means to impact care quality, emphasizing how different factors affect quality of care and how the improvement of care delivery involves targeted interventions at multiple levels of the healthcare delivery system.^33^ The education of medical students may prove to be an excellent target for intervention, especially through the introduction of a cultural patient-partner program.

Future physicians must have a deep understanding of how their own attitudes, perceptions and stereotypes affect their interactions with patients.^11^ Standards established through organizations such as LCME and ACGME guide medical educators in addressing this critical challenge. These standards point to solutions for facing the documented desire by both the Latino population and medical providers to address care disparities experienced by America’s Latino population. Due to LCME and ACGME policies and demographic shifts, medical education programs place increasing emphasis on improving medical students’ cultural competence. Medical school curricula should address knowledge, attitudes and skills related to working with ethnically diverse patients. This challenge is indeed “What Really Matters” as we prepare health care providers for service delivery.

As programs move to incorporate and assess cultural competence, further assessment of social interactions and previous clinical experiences may be warranted, as they may be important predictors of readiness to treat Latino patients. Additionally, the introduction of cultural patient-partner programs at medical schools may be an effective and realistic way to increase student cultural competence within the confines of medical school curricula.

### Acknowledgements

The authors wish to thank Callie Heyne and Spenser Staub for their editorial assistance. The project described was supported by Award Number 1R15CA135349-01A2 from the National Cancer Institute. The content is solely the responsibility of the authors and does not necessarily represent the official views of the National Cancer Institute or the National Institutes of Health.

### Conflict of Interest

The authors declare that they have no conflict of interest.
